# Cancer Stem Cells: Devil or Savior—Looking behind the Scenes of Immunotherapy Failure

**DOI:** 10.3390/cells9030555

**Published:** 2020-02-27

**Authors:** Lorenzo Castagnoli, Francesca De Santis, Tatiana Volpari, Claudio Vernieri, Elda Tagliabue, Massimo Di Nicola, Serenella M. Pupa

**Affiliations:** 1 Department of Research, Molecular Targeting Unit, Fondazione IRCCS Istituto Nazionale dei Tumori di Milano, Via Amadeo 42, 20133 Milan, Italy; lorenzo.castagnoli@istitutotumori.mi.it (L.C.); elda.tagliabue@istitutotumori.mi.it (E.T.); 2 Department of Medical Oncology and Hematology, Unit of Immunotherapy and Anticancer Innovative Therapeutics, Fondazione IRCCS Istituto Nazionale dei Tumori di Milano, Via Venezian 1, 20133 Milan, Italy; francesca.desantis@istitutotumori.mi.it (F.D.S.); tatiana.volpari@istitutotumori.mi.it (T.V.); massimo.dinicola@istitutotumori.mi.it (M.D.N.); 3 Department of Medical Oncology and Hematology, FIRC Institute of Molecular Oncology, Fondazione IRCCS Istituto Nazionale dei Tumori, Via Venezian 1, 20133 Milan, Italy; claudio.vernieri@istitutotumori.mi.it; 4 IFOM, FIRC Institute of Molecular Oncology, via Adamello 16, 20139 Milan, Italy

**Keywords:** cancer stem cells, immunotherapy, tumor microenvironment, immune checkpoint blockade

## Abstract

Although the introduction of immunotherapy has tremendously improved the prognosis of patients with metastatic cancers of different histological origins, some tumors fail to respond or develop resistance. Broadening the clinical efficacy of currently available immunotherapy strategies requires an improved understanding of the biological mechanisms underlying cancer immune escape. Globally, tumor cells evade immune attack using two main strategies: avoiding recognition by immune cells and instigating an immunosuppressive tumor microenvironment. Emerging data suggest that the clinical efficacy of chemotherapy or molecularly targeted therapy is related to the ability of these therapies to target cancer stem cells (CSCs). However, little is known about the role of CSCs in mediating tumor resistance to immunotherapy. Due to their immunomodulating features and plasticity, CSCs can be especially proficient at evading immune surveillance, thus potentially representing the most prominent malignant cell component implicated in primary or acquired resistance to immunotherapy. The identification of immunomodulatory properties of CSCs that include mechanisms that regulate their interactions with immune cells, such as bidirectional release of particular cytokines/chemokines, fusion of CSCs with fusogenic stromal cells, and cell-to-cell communication exerted by extracellular vesicles, may significantly improve the efficacy of current immunotherapy strategies. The purpose of this review is to discuss the current scientific evidence linking CSC biological, immunological, and epigenetic features to tumor resistance to immunotherapy.

## 1. Introduction

Consistent with the concept of cancer immunoediting, many pieces of evidence have underlined the existence of bidirectional crosstalk between cancer cells and cells of innate or adaptive immunity. Specifically, cancer immunoediting, which can constrain or promote tumor development and progression depending on the balance between cancer and immune cells, is a multistep process consisting of different and interchangeable scenarios: 1) the clearance of cancer cells by immune cells, 2) an equilibrium between cancer and immune cells, and 3) the escape phase, with a prevalence of cancer cells over immune cells [1]. During tumor progression, cancer cells acquire specific biological characteristics that lead to immune tolerance, thus preventing or hampering tumor cell attack and killing by antitumor immune cells [2]. In particular, the overexpression of inhibitory immune checkpoints, which impair the anticancer immune response, and/or the release of immunosuppressive cytokines/chemokines are the most common mechanisms that cancer cells utilize to protect themselves from the attack of cytotoxic immune cells [3]. In addition to these mechanisms, genomic instability [4], antigen (ag) loss or downregulation of the ag-presenting machinery [5], the generation of cell hybrids in the tumor microenvironment (TME) [6], the release of extracellular vesicles (EVs) as powerful mediators of intercellular communication [7], and the hierarchical tumor organization arising from cancer stem cells (CSCs) could contribute to immune escape in human cancers [8].

CSCs represent a minor subset of malignant cells capable of unlimited self-renewal and differentiation that contribute to tumorigenesis and tumor aggressiveness, tumor heterogeneity, metastasis, and resistance to antitumor therapies [9,10]. Through asymmetric cell division, a process that underlines the unlimited self-renewal capabilities of CSCs, a single CSC can hierarchically reconstitute the whole cancer cell population, thus regenerating/reseeding the original tumor if implanted in a different organism or in a different site of the same organism; this programme has been defined as “clonal tumor initiation capacity” [9,11]. The ability to shift between different phenotypic cell states by adapting their transcriptome to changes in the surrounding microenvironment confers CSCs the potential to transdifferentiate and invade other tissues and organs, a process also referred to as epithelial-mesenchymal transition (EMT) [12]. Moreover, while cytotoxic agents target the bulk of highly proliferating tumor cells, slowly cycling CSCs can resist chemotherapy and/or radiotherapy, finally resulting in aggressive/advanced treatment-refractory disease [13,14].

Recent studies suggest that CSCs could be crucial players in cancer immune escape; indeed, because of their immunomodulating properties, they can evade immunosurveillance and remain in a quiescent state, thus preventing lethal attack by antitumor immune cells [15,16,17]. Conversely, specific intratumor immune cell populations of the tumor niche interact with CSCs, thus affecting their functional status [18,19]. This biological crosstalk between CSCs and host immunity could represent a new “evil axis” responsible for primary or acquired tumor resistance to immunotherapy, thus paving the way for new therapeutic approaches based on the combination of anti-CSC treatments with immune checkpoint inhibitors (ICIs). In addition, cell–cell fusion, a process that under pathological conditions generates hybrids of tumor cells with diverse types of microenvironmental fusogenic cells, including bone marrow-derived and mesenchymal stem/multipotent stromal cells, macrophages, and fibroblasts, contributes to the formation of aberrant cells with tumor stem cell-like properties associated with tumor initiation, progression, and metastasis [6,20,21]. In general, cell fusion is a genetically regulated process, but external factors, such as hypoxia, inflammation, and mediators of intercellular communication, may also be involved in cell hybrid generation. In particular, it has been reported that EVs, including exosomes, may transport biological cargoes that could to some extent favor cell fusion and the formation of cancer hybrid stem cells that, in turn, promote tumor proliferation, immune evasion, and invasiveness [6]. Notably, EVs have also been found to exert a bidirectional role—EVs from CSCs to the TME and EVs from the TME to CSCs—in different solid cancers, such as breast, colon, lung, and prostate cancers, possibly promoting the development of premetastatic niches [7].

To further complicate this interactive scenario, epigenetic perturbations could also significantly contribute to CSC-related immune escape mechanisms [22]. Indeed, epigenetic modifications of both CSCs and differentiated cancer cells may lead to changes in the gene expression of immune-related genes, which can impact antigen processing/presentation and immune evasion. For instance, demethylating agents may enable re-expression of immune-related genes with potential for therapeutic impact, especially in the setting of combination treatment with immunotherapy [23].

In this review, we aim to highlight specific immunological traits of CSCs, pointing out how an immunosuppressive phenotype can promote tumor aggressiveness, stemness enrichment, and immunotherapy resistance.

## 2. CSCs: Leading Actors in the Intratumor Immunosuppressive Scenario?

The “CSC hypothesis” relies on the existence of a minor population of cancer cells, namely, CSCs, with the peculiar capability to undergo asymmetric divisions, which give rise to another CSC (self-renewal) and, at the same time, to a more differentiated precursor cell that recapitulates a tumor in all its components, including cells with different proliferative and invasive properties. Several studies have demonstrated that CSCs drive cancer cell proliferation, aggressiveness, and recurrence through the activation of specific signaling pathways, such as the Notch, Hedgehog, Wingless (WNT) and NF-kB circuits [22,24]. Notably, CSCs have also been implicated in primary or acquired tumor resistance to radiotherapy, chemotherapy, targeted therapies, and immunotherapy; in particular, CSCs contribute to tumor heterogeneity and metastasis [25]. For these reasons, CSCs may represent the “root of cancer”, and their elimination represents a promising yet investigational therapeutic strategy. To date, the main limitation to the development of effective anti-CSC treatments has been the difficulty of identifying biological markers that univocally characterize CSCs when compared to their normal counterparts.

**Figure 1:**
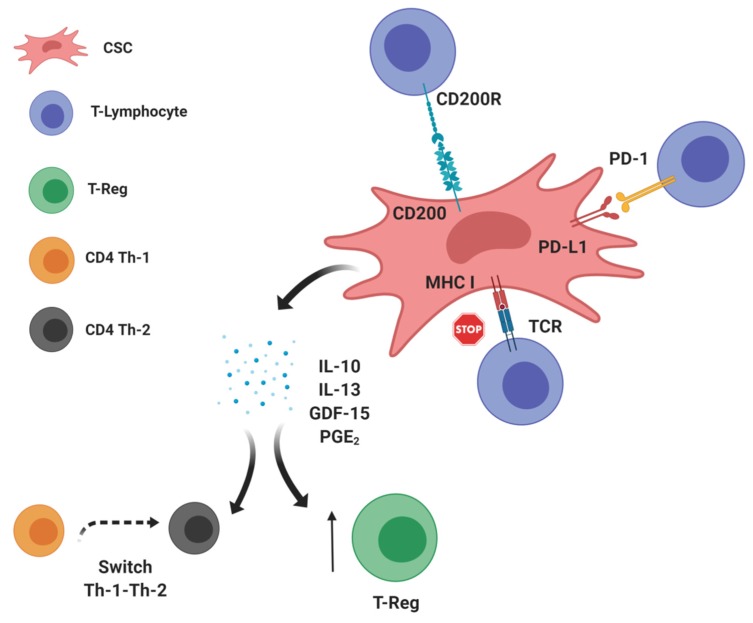
Schematic representing the core findings described in Section Altered expression of molecules involved in ag presentation (MHC I), the activity of immune cells (CD200 and PD-L1), and the release of immunomodulatory molecules with protumorigenic effects (IL-10, IL-13, GDF-15, and PGE_2_) by CSCs. Created by Biorender.

The interplay between CSCs and immune cells is an emerging field of investigation, and different studies have highlighted a key role of CSCs in “re-educating” the immune system, thus modifying the balance between antitumorigenic and protumorigenic immune cells [15,16,17]. The processing and presentation of tumor-associated ags (TAAs) in the tumor cell membrane are two crucial mechanisms driving tumor cell attack by immune cells [26]. TAAs are processed by the cytoplasmic proteasome machinery, transported into the endoplasmic reticulum by TAP1 and TAP2 proteins, loaded onto major histocompatibility complex I (MHC I), and presented to naive CD4+ helper and CD8+ cytotoxic T lymphocytes [27] (Figure 1). Different TAAs expressed by CSCs, such as ALDH1A1, CD133, CEP55, COA-1, EpCam, HEATR1 IL-13Rα2, SOX2, and DNAJB8, are recognized by T cells and are potentially capable of eliciting an effective antitumor immune response [28]. To prevent attack by immune cells, neoplastic cells in clinically detectable tumors often downregulate the expression of proteins involved in ag presentation, thus preventing the exposure of TAAs in tumor cell membranes [29]. This phenomenon appears to be enhanced in CSCs of several cancer types. In particular, a significant decrease in TAP2 and MHC I expression was revealed in the CSC compartment of head and neck and colorectal carcinomas, melanomas, and glioblastomas [30,31,32,33]. The molecular mechanisms responsible for the negative modulation of ag presentation in CSCs are still under investigation [31,33]. Even if the downregulation of members of the ag presentation machinery appears to be a common mechanism used by CSCs to avoid recognition by immune cells, it is not the only mechanism. For instance, studies performed in models of melanoma and glioma revealed no defective expression of HLA class I molecules [34,35]. In fact, CSCs can contribute to an immunosuppressive environment by upregulating the expression of immune checkpoint molecules that impair the activity of immune cells, such as CD200 and PD-L1 (Figure 1). CD200 is a protein expressed on the tumor cell surface, and it is able to promote immunosuppression [36,37]. Indeed, the binding of CD200 on tumor cells with its receptor (CD200R) expressed on T lymphocytes can induce a switch from an antitumor T helper (Th)-1 to an immunosuppressive Th-2 response [38]. CD200 overexpression has been reported in the CSCs of human papillomavirus (HPV)-positive and HPV-negative head and neck squamous cell carcinomas, thus pointing out its candidacy as a potentially actionable CSC marker [39] and paving the way for new therapeutic approaches targeting this immunomodulation-associated protein [40]. PD-L1, an immune checkpoint protein known to trigger a protumorigenic immunophenotype, was also found to be upregulated on the plasma membrane of melanoma as well as ovarian, breast, colon, and lung CSCs [41,42,43,44]. In particular, PD-L1 binding to its receptor (PD-1) on the T cell plasma membrane inhibits the production of immunostimulating cytokines (i.e., IFN-γ and IL-2) [45,46], dampens the T cell-mediated immune response [47], and promotes the expansion of immunosuppressive regulatory T (T-reg) cells [48].

The recent introduction of monoclonal antibodies (mAbs) targeting the PD-1/PD-L1 signaling axis in cancer therapy significantly improved the survival of patients with different advanced and aggressive tumor types, including melanoma, non-small-cell lung cancer, and kidney carcinoma [49]. In agreement with another group [50], we recently showed that the activation of the WNT signaling pathway increased PD-L1 expression on the plasma membrane of triple-negative breast CSCs (TNBCSCs) [42]. We also provided the first evidence that PD-L1+ TNBCSCs interact with tumor-infiltrating CD8+ and PD-1+ immune cells, thus halting antitumor immune responses [42].

In the complex process of immunoediting, a key role is played by cytokines released by the different cell subsets present in the TME. Depending on the type of molecule, different cytokines could promote the killing of cancer cells or, alternatively, they could contribute to establishing an immunosuppressive environment (Figure 1). CSCs can contribute to this mechanism by releasing cytokines that modulate the activity of tumor-infiltrating cells (Figure 1). For instance, melanoma and lung CSCs release IL-10 [32], a proinflammatory cytokine that exerts a negative regulation on T cells and stimulates the expansion of T-reg cells [51]. IL-13, a proinflammatory cytokine that impairs the cytotoxic activity of CD8+ T cells [52], was found to be overexpressed and released by lung CSCs [53]. Moreover, growth differentiation factor (GDF)-15, a member of the transforming growth factor-beta (TGF-β) superfamily of cytokines, was detected in colorectal carcinoma and glioma tumor specimens, where it contributed to promoting EMT, tumor metastasis, proliferation, and immune escape [54,55]. Additionally, GDF15 was found to maintain CSCs in breast cancer tissues by inducing its own expression in an autocrine/paracrine manner [56]. Furthermore, CSCs from glioma and colorectal neoplasms can also produce small molecules with immune-suppressive action. Specifically, the stemness-related WNT signaling pathway is involved in the production of prostaglandin (PG) E_2_ [57], a derivative of arachidonic acid with the capability of inducing a shift from a Th-1 to a Th-2 immune response, and its depletion has been shown to promote antitumor immune attack [58]. Together, these studies indicate the possibility of combining therapies directed against CSCs (by identifying and targeting CSC-related biomarkers) with immune-modulating agents that are already known to promote an effective antitumor immune response. Several ongoing phase I trials are testing the efficacy of WNT (NCT02521844, NCT02675946, and NCT02013154) or Hedgehog (NCT02690948) inhibitors in combination with pembrolizumab, a humanized MAb directed at PD-1.

## 3. Immunomodulatory Molecules Stimulating Tumor Stemness: The Dark Side of CSCs

**Figure 2:**
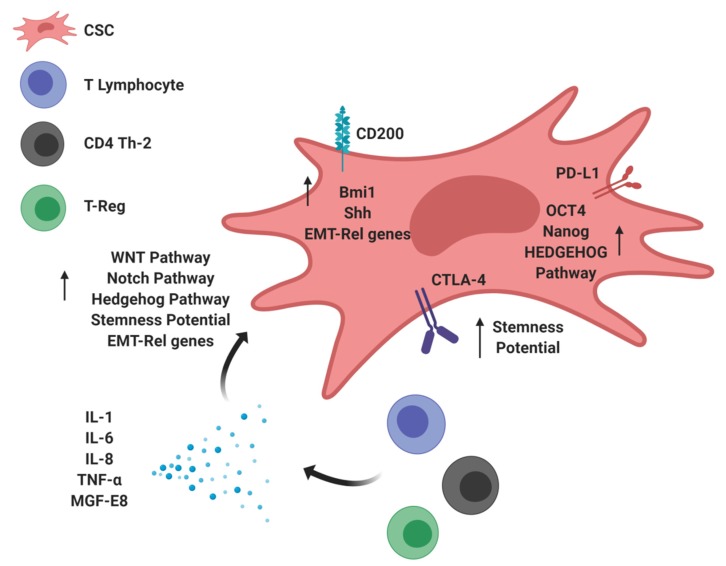
Schematic representing the core findings described in Section Altered expression of molecules involved in intratumor stemness enrichment (CD200, PD-L1, and CTLA-4) occurs via a “direct mechanism”. Dynamic crosstalk occurs between cancer stem cells (CSCs) and immune cells through the release of inflammatory soluble factors (IL-1, IL-6, IL-8, TNF-α, and MGF-E8) by tumor-infiltrating immune cells—a “reverse mechanism”—coupled with the activation of CSC-related signaling pathways (WNT, Notch, and Hedgehog) that sustain the epithelial-mesenchymal transition (EMT) program and CSC maintenance/activity. Created byBiorender.

CSCs are involved in bidirectional crosstalk with protumor or antitumor immune cells. Specific factors that are produced by CSCs to inhibit antitumor immunity also sustain CSC maintenance/activity, thus increasing tumor aggressiveness: a “direct mechanism” from CSCs to immune cells. Conversely, populations of tumor-infiltrating immune cells regulate CSCs by establishing physical interactions with them or through the release of soluble factors, such as cytokines: a “reverse mechanism” from immune cells to CSCs (Figure 2).

Regarding the “direct mechanism”, CD200 expression by CSCs not only inhibits immune system activation (see the previous section) but also sustains stemness enrichment by stimulating the expression of Bmi1 and Shh, two molecules implicated in Hedgehog signaling [59,60] (Figure 2). Moreover, ectopic CD200 expression induces EMT-related genes, enhanced resistance to radiotherapy, and invasiveness in head and neck squamous cell carcinoma and skin tumors [61,62].

PD-L1 expression by cancer cells has also been associated with enhanced stemness potential in different oncotypes. In particular, Almozyan S et al. demonstrated that PD-L1 expression in breast cancer was associated with resistance to chemotherapy and with the induction of EMT [63]. In this study, the authors revealed that PD-L1-silenced breast cancer cells formed mammospheres with significantly decreased efficiency and displayed impaired in vivo tumor-forming capacity when injected in mice in limiting dilution conditions, thus providing evidence for a regulatory role of PD-L1 in CSC self-renewal [63]. In addition, the same authors also reported a direct role of PD-L1 in controlling the expression of the stemness-related factors OCT4, Nanog, and BMI1 [63] (Figure 2). Together, these data indicate that PD-L1 expression in cancer cells not only inhibits antitumor immunity but also may contribute to the maintenance of stemness potential. Consistent with this model, Wei F et al. demonstrated the existence of crosstalk between PD-L1 and the Hedgehog signaling pathway that is mediated through a direct interaction between PD-L1 and HMGA1 [64]. In this study, PD-L1 increased the frequency of intratumor CSCs and contributed to the maintenance of tumor cell self-renewal [64]. Additionally, CTLA4, another immune checkpoint protein that inhibits T cell proliferation, activation, and anticancer immune response [65], was shown to increase the stemness potential in melanoma cells [66] (Figure 2). Indeed, CTLA-4 has a specific role in maintaining stem cell activity, and its inhibition with specific anti-CTLA-4 mAbs suppressed crucial CSC properties, including sphere-forming capability and in vivo tumorigenic potential [66].

Regarding the “reverse mechanism”, soluble inflammatory cytokines released into the TME by tumor-infiltrating immune cells could modulate CSC activities as well. For instance, the soluble factor IL-1 promoted Th-1 to Th-2 differentiation, thus contributing to the inhibition of antitumor immunity [67,68]. In particular, IL-1β stimulated the expression of the CSC-related marker ZEB1, induced resistance to chemotherapy, and increased sphere-forming efficiency in colon cancer cells, thus supporting a role for IL-1β in promoting CSC self-renewal and EMT [69]. In pancreatic cancer, KRAS-driven signaling activates an intrinsic inflammatory programme by activating the CSC-related NF-κB signaling pathway, which in turn stimulates the production of cytokines of the IL-1 family that promote early tumor invasiveness, EMT, angiogenesis, and metastasis [70]. Other proinflammatory cytokines, such as IL-6 and IL-8, could increase the number and activation of specific CSC subpopulations. In particular, IL-6 can act intrinsically on tumor cells, but it can also stimulate antitumor adaptive immunity [71]. IL-6 stimulates the expression of genes involved in stemness, invasion, migration, and tumorigenesis through a mechanism that involves its interaction with STAT3 [72,73]. IL-6 also causes resistance to chemotherapy, a process strongly linked to CSC enrichment, in different tumor types, including renal and ovarian carcinomas [74,75]. IL-8, a proinflammatory cytokine able to convert the anticancer immune response into an immunosuppressive response through its binding with CXCR1/2 receptors [76], is also involved in the acquisition and/or maintenance of stemness features in lung, renal, pancreatic, and breast cancers [77,78]. In particular, the inhibition of the IL-8/CXCR1/2 axis efficiently targets the CSC compartment in pancreatic cancer [78], and the combination of repertaxin, an antagonist of the CXCR1 receptor, with lapatinib, a tyrosine kinase inhibitor that halts HER2-mediated signaling, impairs mammosphere formation by HER2-positive cancer cells more efficiently than repertaxin or lapatinib monotherapy [79]. In this complex scenario, tumor necrosis factor (TNF)-α, a potent proinflammatory cytokine with putative suppressive effects in antitumor immunity and that has been reported to be involved in the expansion of T-reg cells [80], is implicated in the regulation of CSC-related features. In particular, TNF-α promotes cancer invasion and metastasis associated with the EMT programme in colorectal cancer [81]. Furthermore, milk fat globule epidermal growth factor-8 (MGF-E8), a secreted mediator of local immune suppression that stimulates T-reg cell infiltration and suppresses the Th-1 response and NK cell and CD8+ T cell cytotoxicity [82], was also found to enhance tumor cell survival, invasion, and angiogenesis and to modulate CSC activity. Specifically, in colon cancer, MGF-E8 released by TAMs induces the activation of the Hedgehog pathway, driving melanoma progression [83]. All these considerations indicate that an immunosuppressive TME not only affects the function of intratumor immune cells but also has an impact on CSC number and activation status.

## 4. Epigenetic Control of Stemness Features and Immune Escape

Among the mechanisms regulating stemness properties, epigenetic mechanisms play a pivotal role by shaping the plasticity and biological behaviour of CSCs. Epigenetics refers to the set of dynamic changes in gene expression and cellular phenotypes that occur in the absence of genomic modifications. In recent years, the study of epigenetics has attracted much interest because epigenetic modifications have been shown to heavily affect tumor cell fate [84]. The main epigenetic events that contribute to cancer development consist of aberrant DNA methylation, histone modifications, chromatin remodeling, and changes in noncoding RNAs, including miRNAs. In particular, the discovery of histone post-translational (hPT) modifications led to the so-called “histone code hypothesis”, according to which a dynamic constellation of biochemical modifications determines the binding of chromatin-remodeling factors to the nucleosome, a region of DNA wrapped around eight histone protein cores [85,86]. By altering chromatin structure, chromatin-remodeling factors regulate DNA accessibility by transcription factors, co-factors, and the general transcription machinery, thus modulating gene expression in cancer cells. These biological processes govern the epigenome landscape, which represents a central element during cancer progression and the expression of stemness properties. In this context, breakthrough studies by McCullough and Weismann have described the epigenetic-driven hierarchical organization of the hematopoietic system, with leukemia representing a paradigm governed by plastic leukemic stem cells [87,88]. Specifically, different studies addressing the link between stemness phenotypes and epigenetic control have analyzed the formation of the LSC compartment in acute lymphoblastic leukemia and acute myeloid leukemia. These studies highlighted the presence of chromosomal rearrangements involving KTM2A/MLL, which encodes a modified histone methyltransferase that is able to constitutively activate DNA transcription. It is evident how the establishment of epigenetic aberrations serves as an early event leading to the accumulation of cells with increased stemness properties and self-renewal ability.

Among brain neoplasms, glioblastoma represents the most aggressive and deadly type and is characterized by high histologic grade, an undifferentiated phenotype, and a high frequency of CSCs. Several studies have identified gain-of-function mutations in genes encoding histone H3 in pediatric high-grade gliomas, with at least two recurrent mutations within the histone H3.3 gene H3F3A identified in nearly 50% of the patients [89,90]. These two mutations result in the substitutions of the K27 or G34 residue with a methionine (K27M), an arginine (G34R), or a valine (G34V) residue. Of note, the K27M substitution causes the inhibition of polycomb repressive complex 2, with a genome-wide reduction in H3K27me3 and the re-establishment of an earlier developmental program in neural precursor cells and the reacquisition of oncogenic self-renewal ability [91].

In breast cancer, key CSC-related gene pathways are finely regulated by epigenetic mechanisms and their inhibition affects cancer cell survival, self-renewal, tumorigenesis, and progression [92]. For instance, epigenetic modifications regulate genes of the Notch and WNT pathways, which control normal mammary development and breast cancer self-renewal [93]. Moreover, nuclear receptor corepressor 2, which recruits histone deacetylases (HDACs) to the promoter regions of Notch target genes (e.g., MYC and HES1) to regulate their transcription [94], has been found to be upregulated as a result of reduced recruitment of corepressor complexes with chromatin-remodeling activity, finally resulting in enhanced transcription of Notch target genes [95]. In a variety of human solid malignancies, including colorectal cancer, hepatocellular cancer, and breast cancer [96], evidence is accumulating that epigenetic mechanisms promote tumorigenesis by leading to the activation of the WNT/β-catenin pathway. For instance, DNA methylation and histone modifications of WNT regulators, such as WNT inhibitor factor 1, AXIN2, secreted frizzled-related protein 1, and Dickkopf-related protein 1, have been frequently reported in breast cancer and are responsible for aberrant WNT/β-catenin gene pathway expression/activation [97]. Moreover, some WNT proteins, such as WNT1, WNT2, WNT3A, and WNT5A, which are frequently overexpressed in breast, colon, lung, and prostate cancers as a result of epigenetic modifications, behave as oncogenic activators of the canonical WNT signaling pathway [98]. Because aberrant activation of the WNT/β-catenin axis contributes to cancer progression, the identification of epigenetic events associated with WNT/β-catenin activation could provide useful biomarkers for cancer detection and prognosis.

**Figure 3:**
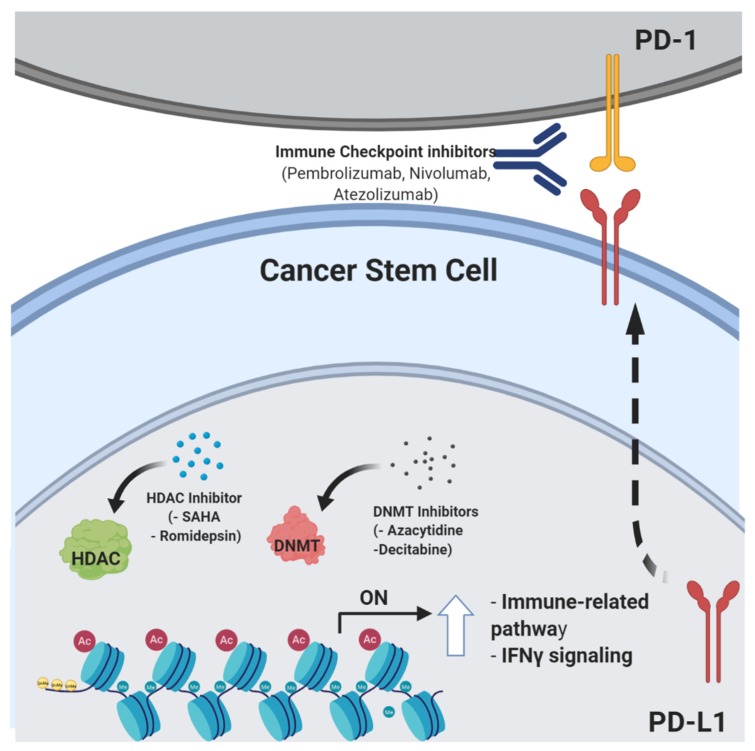
Schematic representing several epigenetic modulators (histone deacetylases (HDAC) and DNA methyltransferase (DNMT) inhibitors) that may act by sensitizing tumor cells to immune checkpoint blockade. Created by Biorender.

Specific epigenetic events taking part in tumor progression are also involved in tumor immune escape. For instance, different studies, reviewed in [99], have identified mutations or structural defects in human and mouse genes that are known to be epigenetically regulated in CSCs and are involved in the control of T and B cell differentiation [100,101,102]. Moreover, epigenetic modulators have been shown to restore functional immune cell recognition and immunogenicity, providing a proof-of-concept demonstration that targeting tumor epigenetics could have synergistic antitumor activity with currently available immunotherapy strategies (Figure 3) [103,104,105].

Taken together, published data indicate that bulk tumor cells and CSCs may exploit epigenetic repression of specific immune genes to escape killing by immune cells. It is likely that the interplay between genetic and epigenetic modifications affects the carcinogenesis process and takes part in the selective pressures involved in immune cell escape. This has been clearly demonstrated in an analysis of several melanoma cell lines derived from patients who underwent successful immunotherapy and recurrence [106]. The scientific rationale for the use of HDAC inhibitors (HDACi) or DNA methyltransferase inhibitors (DNMTi) in ongoing clinical trials in cancer patients relies on the ability of these drugs to inhibit tumor cell growth and to induce cell differentiation [107]. However, DNMTi and HDACi are also capable of upregulating the expression of TAAs in different solid tumors [108,109,110,111], thus potentially sensitizing cancer cells to ICIs by upregulating CTLA4, PD-1, PD-L1, and PD-L2 molecules on both tumor cells and TILs [112,113]. HDACi can also restore the expression of MHC class I and II molecules, which are downregulated as a result of transcriptional alterations in the IFNγ and NF-kB (for MHC class I) or the CIITA (for MHC class II) pathways [108], thus facilitating innate and adaptive antitumor immune responses. Other studies have linked HDACi to both increased and decreased expression of PD-L1 and PD-L2 on tumor cells [114,115]. However, the great potential for epigenetic-targeted interventions lies in the fact that, unlike genetic abnormalities, epigenetic modifications are reversible, thus allowing fast and potentially complete functional restoration of otherwise intact genes [113,116]. An exciting advance towards the combination of epigenetic and immune therapies is the fact that epigenetic therapy may reverse immune tolerance in human cancers [115,116]. Clinical trials are ongoing to explore this hypothesis. This kind of combination therapy may significantly benefit from the advent of next-generation sequencing (NGS)-based approaches, which massively amplify the possibility of studying new strategies, with the goal of achieving successful precision medicine and overcoming resistance to immunotherapy [117]. In particular, these techniques are based on the analysis of the epigenome, defined by a set of chemical modifications, such as methylation and acetylation of DNA and/or DNA-binding histone proteins, and include chromatin immunoprecipitation (ChIP) assays coupled to NGS, commonly known as ChIP-seq, and methylation analysis through whole-genome bisulfite/array-based sequencing. By harnessing the power of NGS, it is possible to analyze genome-wide DNA-binding sites for transcription factors and histone proteins and methylome patterns at a single-nucleotide resolution [118].

## 5. Conclusions

In this review, we have discussed the growing body of current scientific evidence linking CSC biological, immunological, and epigenetic features to tumor resistance to immunotherapy. A better understanding of the molecular mechanisms regulating the dynamic interplay between cells in the TME and CSCs could pave the way for future anticancer therapies aimed at targeting CSCs by reversing intratumor immunosuppression.
